# Interactions of Nanomaterials with Gut Microbiota and Their Applications in Cancer Therapy

**DOI:** 10.3390/s23094428

**Published:** 2023-04-30

**Authors:** Xiaohui Li, Huan Wei, Jiachen Qi, Ke Ma, Yucheng Luo, Lixing Weng

**Affiliations:** 1 School of Geography and Bioinformatics, Nanjing University of Posts and Telecommunications, Nanjing 210023, China; lixh@njupt.edu.cn (X.L.);; 2 College of Materials Science & Engineering, Nanjing University of Posts and Telecommunications, Nanjing 210023, China

**Keywords:** nanomaterials, gut microbiota, nanotechnology, cancer therapy

## Abstract

Cancer treatment is a challenge by its incredible complexity. As a key driver and player of cancer, gut microbiota influences the efficacy of cancer treatment. Modalities to manipulate gut microbiota have been reported to enhance antitumor efficacy in some cases. Nanomaterials (NMs) have been comprehensively applied in cancer diagnosis, imaging, and theranostics due to their unique and excellent properties, and their effectiveness is also influenced by gut microbiota. Nanotechnology is capable of targeting and manipulating gut microbiota, which offers massive opportunities to potentiate cancer treatment. Given the complexity of gut microbiota–host interactions, understanding NMs–gut interactions and NMs–gut microbiota interactions are important for applying nanotechnologies towards manipulating gut microbiota in cancer prevention and treatment. In this review, we provide an overview of NMs–gut interactions and NMs–gut microbiota interactions and highlight the influences of gut microbiota on the diagnosis and treatment effects of NMs, further illustrating the potential of nanotechnologies in cancer therapy. Investigation of the influences of NMs on cancer from the perspective of gut microbiota will boost the prospect of nanotechnology intervention of gut microbiota for cancer therapy.

## 1. Introduction

Cancer is a major cause of death worldwide. Research in nanotechnology elicits advances in the development of miscellaneous nanomaterials (NMs) for cancer treatment [1]. NMs can be engineered with unique structures and functions, providing novel tools and techniques [2]. Indeed, NMs have led to several promising applications in cancer diagnosis and treatment, including drug delivery, diagnostic imaging, synthetic vaccine development, and miniature medical devices [1]. Although innumerable preclinical studies have been announced with preliminary triumphs, cancer nanomedicine has to face multiple obstacles before being adopted as a safe option for cancer patients in clinical practice [3]. These obstacles are largely attributable to the complexity of interactions between NMs and the living organisms. There are multiple biological steps in the systemic or local delivery of NMs, such as NM–protein interaction, extravasation into and interaction with the perivascular tumor microenvironment (TME), tumor tissue penetration, and tumor cell internalization [4,5], all of which can influence the effect of NMs. In turn, nanoparticles (NPs) properties (for example, size, surface features, composition, and targeting ligand) can influence these biological processes, thus determining the enhanced permeability and retention (EPR) effect and therapeutic outcomes [6]. So, it is of vast significance to explore the biological effects of NMs. However, the interaction between NMs and the organisms is so complicated that their potential interactions and effects on each other have not yet been thoroughly investigated [7].

Generally, NMs can be delivered into individual organisms through different administration routes, exerting varying degrees of impact on organs, organelles, tissues, and cells [8]. When entering into the gut lumen, NMs inevitably get in touch with gut microbiota [9]. Gut microbiota is a diverse microbial ecosystem [10] and plays a crucial role in an individual’s health status [11]. Considerable evidence has demonstrated the tight association between gut microbiota and tumorigenesis, as well as gut microbiota and anticancer therapies [12,13]. Gut microbiota is an essential mediator of the positive effects of NMs. There is evidence that successful modulation of gut microbiota can augment therapeutic efficacy for cancer by nanotechnology [14]. In addition, nanotechnology is also an opportunity to advance the understanding of the interaction between NMs and the organism through ongoing research on gut microbiota and NMs in cancer. The incomplete understanding of nano–biological systems interactions is one of the major obstacles to the advancement of cancer nanomedicines, but it also contains many opportunities. Timely review and summary of the interactions between NMs and gut microbiota and their applications will be conducive to diversified innovations in nanotechnology and cancer therapeutics. Therefore, this review attempts to consider gut microbiota and nano–bio interactions as they are relevant to driving innovation in cancer treatment and clinical transformation of cancer nanomedicines. We first provide an overview of our understanding of NMs–gut interactions, and then discuss NMs–gut microbiota interactions and their impact on the therapeutic effect of NMs. Lastly, we shed light on the application and feasibility of nanotechnology intervention in gut microbiota for cancer therapy. We believe that this review can provide support for basic research and clinical applications in this field.

## 2. Interaction between NMs and Gut

**Figure 1:**
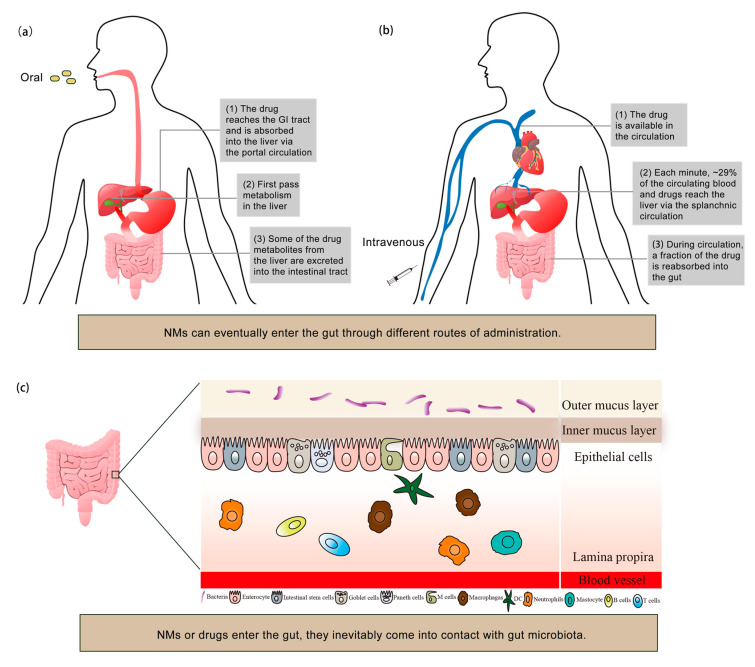
The route of NMs entering the gut and its interaction with the gut. (**a**) Orally administered drugs or NMs into the gut. (**b**) Intravenously administered drugs or NMs into the gut. (**c**) Basic structure of the gut.

The nanodrug systems composed of NMs and nanostructures can achieve a variety of anticancer functions, including targeted drug delivery, stimulus response, and treatment [15]. There are various administration routes in the process of exerting these anticancer functions of nanodrug systems [16]. Figure 1a,b presents a schematic representation of drug transport routes within the body following enteral (e.g., oral) or parenteral (e.g., intravenous) administration. Regardless of the route of administration, NMs can enter the gut and further interact with each structural part of the gut. The fundamental structure of the gut includes the gut lumen, the mucus layer, the epithelial cell layer, and the lamina propria, as well as blood vessels (Figure 1c). Trillions of gut microbiota inhabit the mucous layer and permeate the various functional parts of gut by metabolites interacting with immune cells and their secreted factors. When NMs enter the gut, they inevitably come into contact with gut microbiota. According to the current literature, gut microbiota is considered to be the determinant in the therapeutic efficacy or toxic side effects of NMs by participating in many fundamental biological processes [17]. Therefore, it is necessary to deeply explore the interactions between NMs and gut microbiota.

## 3. Interactions between NMs and Gut Microbiota

Although the potential implications of gut microbiota–NMs interactions remain largely unexplored, current knowledge underlines their complexity and bidirectionality—NMs influence the structure and/or metabolic activity of gut microbiota; in turn, gut microbiota affects the bioactivity or toxicity of NMs [18].

### 3.1. Influences of Gut Microbiota on NMs

Existing studies on pharmacokinetics (e.g., metabolism, enzyme degradation) and pharmacokinetics (e.g., immune regulation) have indicated that the impact of gut microbiota on anticancer drugs is extremely complex [19,20]. The gut microbiota participates in the biotransformation and metabolism of anticancer drugs by enzymatic reactions, and further alters the structure, bioavailability, biological activity, or toxicity of drugs [21,22]. A variety of anticancer drugs, such as irinotecan [23], cyclophosphamide [24], 5-fluorouracil [25], etc., can be metabolized by gut microbiota, accompanied by the production of a large number of small molecules, such as butyric acid and bile acid [23,26]. These metabolites are finally decomposed into active, inactive, or toxic substances after complex interactions with the organisms [27]. In addition, these active or toxic substances have positive or negative influences on host immune responses to cancer therapies. For example, the gut microbial metabolite butyrate has been known to modulate CD8^+^ T cell function in the tumor microenvironment and boost the efficacy of oxaliplatin-based chemotherapy [28].

Compared with traditional drugs, nanodrugs, characterized by regulating solubility, permeability, and bioavailability, have greater efficiency and less toxicity in cancer treatment. Meanwhile, modified nanodrugs can prolong their residence times in the gastrointestinal tract, which may improve their interactions with gut microbiota for biotransformation [29]. Thus, it has become increasingly important in the significance of gut microbiota as a key variable of the pharmacodynamic and corresponding cancer therapeutic response to nanodrugs. There is evidence that insoluble metal oxides, such as CdO, CuO, PbO, and ZnO, can be dissolved directly or indirectly by anaerobic microorganisms through enzymatic activity or microbial metabolites [30]. Metal NMs can also interact with anions in intestinal mucus by releasing metal cations for mucus-targeted therapy [31]. For example, natural polymers (such as hyaluronic acid, chitosan, and pectin) and synthetic polymers (such as acrylic derivatives) have been found to nonspecifically adhere to mucins [32]. Given the enormous latent capacity of gut microbiota for drug metabolism and transformation, it is reasonable to speculate that the peculiarities of ingested NMs are altered as they pass through the digestive tract and interact with the gut environment. Gut microbiota is a key factor affecting the biological effects of nanodrugs.

### 3.2. The Effect of NMs on Gut Microbiota

Gut microbiota, as an orderly and complex ‘micro-ecosystem’ [33], performs a variety of functions through extensive interactions with the host [34]. Once the gut microbiota is disrupted, it has a profound impact on host’s health [35]. Omics studies have found that NMs at sub-growth-inhibitory or sub-toxic concentrations can meddle in bacterial physiology and function, involving many pathways, such as membrane transport and signal transduction, central and energy metabolism, quorum sensing, or biofilm formation [30]. The above pathways are vital for bacteria to survive and thrive. Thus, perturbation of these pathways potentially alters the composition, diversity, and abundance of gut microbiota. In vivo experiments reveal that many NMs, such as Gold NPs, Ag NPs, Titanium Dioxide NPs, and single carbon nanotubes (SWCNTs), can disturb the structure and/or function of gut microbiota by altering their diversity (Table 1), and the magnitude of the effect depends largely on intrinsic properties of NMs, such as size, shape, composition, and surface chemistry [34,36,37].

Gut microbiota dysbiosis is typically characterized by a reduction in the number of beneficial bacteria and microbial diversity [38], which may trigger inflammatory signaling pathways with effects that span far beyond the level of the gut [39]. In fact, NMs can disrupt gut homeostasis and elicit dysbiosis of gut microbiota, leading to local or systemic inflammatory responses [40]. Specifically, NMs exposure may cause inflammatory responses by inducing a decrease in the anti-inflammatory *firmicutes* or an increase in the pro-inflammatory *bacteroidetes* in gut [41]. In addition, such local effects may be developed into systemic effects though multiple pathways, such as translocations of gut microbiota or bacterial products and toxins or metabolites circulating in blood [42]. To investigate the effects of NMs on gut and gut microbiota, BALB/c mice were administered different doses of intravenous cadmium telluride quantum dots (CdTe QDs). The results suggest that changes in gut microbiota are implicated in the effects of CdTe QDs on the gut and liver immune systems and lipid metabolism [43]. Indeed, gut microbiota plays a key role in regulating gut inflammation, liver immunity, and lipid metabolism. For instance, bile acid is converted into immune signaling molecules by gut microbiota to regulate immunity and inflammation [44,45,46].

**Table 1:** The changes in abundance of major gut bacterial phyla after exposure to NMs in mice.

NMs (Size)	*Bacteroidetes*	*Firmicutes*	*Proteobacteria*	Exposure	Time (Day)	Concentration	Ref
TiO_2_ NPs (rutile, 16 nm) ^1^	down	up	-	Gavage	28	100 mg/kg/day	[47]
SiO_2_ NPs (11 nm) ^1^	down	up	up	Gavage	7	2.5 mg/kg/day	[48]
Ag NPs (12 nm) ^1^	up	down	up	Gavage	7	2.5 mg/kg/day	[48]
HAHp/ZnO NPs (10–40 nm) ^1^	down	up	down	Gavage	14	1000 mg/kg/day	[49]
Ag NPs (PVP, 55 nm) ^1^	down	up	down	Diet	28	0.04, 0.46, 4.6 mg/kg	[50]
SWCNTs (1.1 nm × <5 μm) ^2^	up	down	up	Diet	90	15 or 150 mg/kg feed	[37]
MWCNTs (20 nm ×< 2 μm) ^2^	up	down	up	Gavage	28	0.001, 0.01, 0.1 mg/day	[37]
MWCNTs (<74 nm × <5.7 μm) ^2^	-	-	-	Gavage	7	2.5 mg/kg/day	[51]
Graphene (300–2000 nm) ^3^	down	down	down	Gavage	28	0.001, 0.01, 0.1 mg/day	[52]
MoS_2_ (20–1000 × 1–10 nm) ^3^	down	up	-	Diet	90	15 or 150 mg/kg feed	[53]
GO (0.8–14.3 μm^2^ × 1nm) ^3^	down	up	down	Gavage	7	2.5 mg/kg/day	[37]

^1^ 1D NMs; ^2^ 2D NMs; ^3^ 3D NMs. ^2, 3^ blateral size × thickness.

Immunity is one of the core hubs for gut microbiota to modulate various physiological systems (gut-heart axis, gut-lung axis, gut-liver axis, etc.) [54]. The alterations in the gut microbiota–immune system axis are associated with many chronic diseases [55], such as inflammatory bowel diseases (IBDs) [56]. Although their etiology remains largely unknown, numerous studies have demonstrated that the pathogenesis of IBDs is linked with dysbiosis of gut microbiota and dysregulated intestinal immune responses [57,58]. NMs have the capability to treat IBDs. Multitudinous nanoscale drug-delivery systems are used to improve IBD outcomes [59]. A previous study has shown that tungstate treatment can downregulate the explosive growth of facultative anaerobes *Enterobacteriaceae* in mouse models of colitis by selectively inhibiting molybdenum-cofactor-dependent microbial respiratory pathways [60]. A study in which C57BL/6J mice were exposed to tungsten oxide (WO_3_), NPs have shown that WO_3_ NPs exhibited a significant therapeutic efficacy against dextran sulfate sodium (DSS)-induced IBD by remodeling gut microbiota homeostasis and significantly reducing intestinal inflammation [61]. Long-term chronic inflammation has been recognized as a key risk factor for colorectal cancer (CRC). Strategies to regulate gut microbiota have great application potential for the prevention and treatment of tumors [62]. Tungsten selenide nanosheets (WSe_2_ NSs) further demonstrated the preventive ability against colitis-associated CRC by targeting *Enterobacteriaceae* in the azoxymethane (AOM)/DSS mouse model of colon carcinogenesis (unpublished work). Exosomes are another classic example. Exosomes from different sources have the ability to reshape gut microbiota and regulate host immunity, thus alleviating disease [63,64,65,66]. These works have demonstrated that the unique physicochemical properties of NMs can be utilized to rationally design microbiota-editing approaches for improving dysbiosis-associated inflammation and even cancer.

Overall, the interaction between NMs and gut microbiota is mutual. Some NMs can perturb gut microbiota and lead to various gut microbiota-related intestinal diseases, such as enteritis and CRC. However, harnessing distinctive features of NMs can also restore the balance of gut microbiota to alleviate and treat diseases. Given the complex involvement of gut microbiota in the biotransformation of NMs, appropriately designed NMs to modulate gut microbiota have great therapeutic potential against diseases. To fully exploit the therapeutic potential of NMs, the influences of gut microbiota on the therapeutic effect of NMs need to be further discussed.

## 4. Influence of Gut Microbiota on the Therapeutic Effect of NMs

### 4.1. NMs Exert Anti-Tumor Effects by Enhancing Immunity

**Figure 2:**
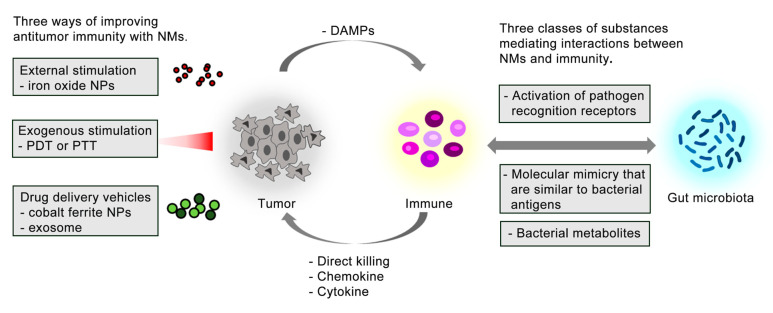
NMs exert anti-tumor effects by enhancing immune function in three ways. Gut microbiota mediates the interaction with immunity through three types of substances, affecting the effect of NMs on immune system treatment of cancer. DAMPs—damage associated molecular patterns.

Malignant tumors are hard to defeat because of recurrence and metastasis after treatment [67]. In theory, effectively stimulating and mobilizing the immune system can provide new opportunities for cancer treatment. Research shows that immune infiltration affects the prognosis of cancer patients [68]. Specific immune components are connected with positive prognosis in most cancers, such as natural killer (NK) cells, M1 macrophages, CD8^+^ T cells, and T follicular helper (TFH) cells. Conversely, macrophages (especially M2), T helper cell 17 (TH17), regulatory T (T_reg_) cells, and polymorphonuclear myeloid-derived suppressor cells (PMN-MDSCs) are mostly indicative of the negative prognosis. In practice, engineered NPs have immune-modulatory properties and can be modified to improve immune responses and enhance antitumor effects [69]. NPs can be divided into three categories according to the way of enhancing tumor immunity (Figure 2). First, some natural or synthetic NMs have functions in immunity regulation without external stimulation [70]. For example, iron oxide NPs could consolidate the macrophage-modulated cancer immunotherapies [71]. Second, NMs can trigger immunogenic cell death and enhance immune responses upon exogenous stimulation, such as photodynamic therapy (PDT) or photothermal therapy (PTT) [72,73]. Reactive oxygen species (ROS), as a main molecule in killing tumor cells, is produced synergically by NPs and exogenous stimuli [74]. When cancer cells are induced by ROS to die via necrosis, apoptosis, autophagy, etc., they will synthesize and release a series of immune effectors. Tissue macrophages and dendritic cells (DCs) first respond, mature, and then activate multifaceted and complex immune responses [75]. This process is supported by experiments [76,77]. Compared with endogenous responses that rely on the characteristics of the tumor microenvironment, exogenous stimuli-responsive NMs can more effectively enhance immune responses at tumor sites to lead to more precise and controllable treatments. Furthermore, NMs are utilized as drug delivery vehicles for transporting other immune stimulatory molecules to specific immune cells or tumor sites. Some immune stimulatory molecules (e.g., chemical drugs, photosensitizers, proteins, plasmids, etc.) are difficult to trigger an adequately strong immune response to inhibit tumor growth. Multifunctional NPs can be designed to more safely and effectively enhance the antitumor response due to their superior functions, such as high loading, targeted delivery, and controllable release [78]. For example, several drug delivery systems based on cobalt ferrite NPs or exosome have been successfully developed and applied in cancer [79,80].

To sum up, NMs as a platform for immunomodulation can boost the immune response. Although remarkable achievements have been made in nanocarrier-based cancer treatments, preparations of NMs with outstanding properties, and designs of novel nanocarriers to evoke specific immunological responses and improve delivery efficiency should be focused in future research. The development of these innovative therapeutic NMs offers a grand opportunity for cancer treatment.

### 4.2. Gut Microbiota Influences the Effects of NMs on Cancer Treatment by the Immune System

Considering the complex gut microbiota–immunity interaction (Figure 2), the immunity-enhancing effects of NMs in cancer are under the interference of gut microbiota [81,82,83]. In mouse models, cyclophosphamide (CTX) induces the translocation of specific gram-positive bacteria into lymphoid organs and promotes the generation of tumor suppressor T helper 17 (“pathogenic” T helper 17, pTH17), while germ-free mice or mice pretreated with antibiotics (ABX) develop resistance to CTX [24]. These results suggest that gut microbiota has a significant impact on cancer therapy by manipulating the anticancer immune effects of CTX. Similarly, another study confirmed that tumor-infiltrating myeloid-derived cells were regulated by gut microbiota, and that disruption of gut microbiota by treatment with ABX directly impaired the host’s response to CpG-oligonucleotide-mediated immunotherapy and platinum-based chemotherapy for subcutaneous tumors [84]. Further, gut microbiota responds correspondingly to the inflammatory tension required by different therapeutic protocols. There are subsets of gut microbiota that activate immunity and subsets of gut microbiota that mediate immunosuppression [85]. Particularly, specific microbiota has been highlighted to play significant roles in an increasing number of studies on cancer immunotherapy. Thus, precision editing of gut microbiota can help reshape the immune signature in cancer to improve the efficiency of cancer therapy [86,87]. *Bifidobacterium* can mediate T cell-enhancing PD-1-based antitumor immune responses by directly leading to DC maturation and cytokine production [84]. *Bifidobacterium pseudolongum*, *Lactobacillus johnsonii,* and *Olsenella* species have also been investigated to assist immunotherapy to achieve tumor suppression or ablation [88]. The ability of *Bifidobacterium* to enhance the response to immunotherapy is largely attributable to the systemic transport of *B. pseudolongum*-derived inosine (A_2_A) to enhance T cell-specific responses. A_2_A receptors are expressed in T cells. This, again, indicates the importance of specific gut microbiota in regulating antitumor immunity and the necessity of accurately editing gut microbiota.

Taken together, strategies to potentiate the immune system are a topic of intensive research and development in cancer treatment. Gut microbiota can influence antitumor immunity through multiple pathways, and the efficacy of cancer therapy depends on the existence of specific gut microbiota. NMs are capable of interfering with gut microbiota to enhance antitumor immunity. Accordingly, enabling NMs to adjust specific microbial species and immune cells will be a future development direction for cancer therapy. However, the dynamic ecosystem of gut microbiota and the complexity of its interactions with the host’s immunity impose a certain challenge to research in this area. Considering the success of nanotechnology in cancer treatment, there are massive opportunities at the intersection of nanotechnology, gut microbiome, and cancer.

## 5. Intervention of Gut Microbiota Based on Nanotechnology to Improve the Effect of Cancer Treatment

**Figure 3:**
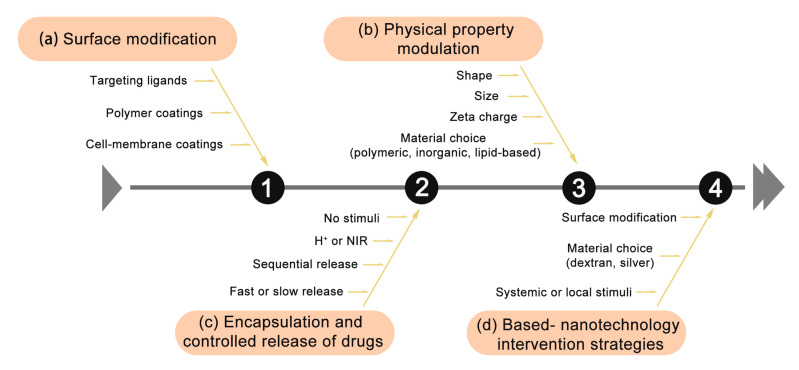
Strategies for improving NMs properties include: (**a**) surface modification, (**b**) encapsulation and controlled release, (**c**) physical modulation, and (**d**) microbiome intervention for cancer therapy based on the above approaches.

The strategies for intervening gut microbiota include regulating commensal microbiota, increasing beneficial bacteria, or decreasing pathogenic flora [89]. Particularly, the specific microbial species we emphasized above could be regulated to improve the pathological conditions to a large extent because they normally play an important role in diseases. However, as a system, gut microbiota is interlinked and dynamically balanced. So, the regulation of gut microbiota requires the capabilities of precise targeting and editing, which is a key challenge for nanotechnology. Fortunately, several sensing technologies based on fluorescent probes are being developed to visualize the distribution of gut microbiota in mice or quantitatively assess functional changes in specific microbial species [90,91,92]. Nanotechnology further enables targeted drug delivery and microbial intervention by the nanotechnology toolbox (Figure 3) [81]. The capacities of various nanodrugs systems to regulate the gut microbiota as anticancer platforms have been reported [93,94]. An example is prebiotics dextran-encapsulated probiotic spores by *Clostridium butyricum* (*C. butyricum*). Glucan was fermented by *C.butyricum* to produce short-chain fatty acids (SCFAs), and they significantly raised the abundance of SCFA-mediated bacteria (such as *Eubacterium* and *Roseburia*) in the gut, which dramatically increased the overall richness of gut microbiota and markedly suppressed colon cancer [95]. Targeted approaches can modulate gut microbiota to an anticancer state to enhance tumor lethality. The work accentuates the feasibility of using a high-security approach to modulating gut microbiota, and it furnishes an auspicious tactics for treating miscellaneous gastrointestinal diseases. In addition to targeting gut microbiota and remodeling the gut microenvironment, rationally designing nanosystems can also reshape the tumor immune microenvironment. A phage-based irinotecan–dextran hybrid nanosystem utilizes dextran to promote the proliferation of *C. butyricum*, an oncogenic bacterium, can selectively increase immunosuppressive myeloid-derived suppressor cells (MDSCs) to hinder the host’s anticancer immune response), thereby rebuilding the antitumor immune microenvironment and inhibiting the development of CRC [96]. These studies demonstrate the great opportunities for gut microbiome-targeted approaches by nanotechnology for cancer prevention and treatment. Actually, gut microbiota-targeted avenues can also be utilized to treat other metabolic and mental health disorders linked with gut dysbiosis, such as obesity and Alzheimer’s disease [97]. Therefore, strategies based on nanotechnology to intervene the gut microbiota deserve greater attention and more in-depth research.

In addition to the gut microbiota, the intratumoral microbiota has also attracted increasing attention [98]. These intratumoral microbiota can intervene local tumor progression independently of the gut microbiota and are unique in different cancers [99]. This finding provides another possibility for manipulating bacteria to potentiate anticancer efficacy. In conclusion, nanotechnology for intervening microbiome to induce antitumor responses has potential applications in tumor therapy, and the intervention methods exist in gut microbiota or tissues containing intratumoral microbiota.

## 6. Conclusions and Perspectives

**Figure 4:**
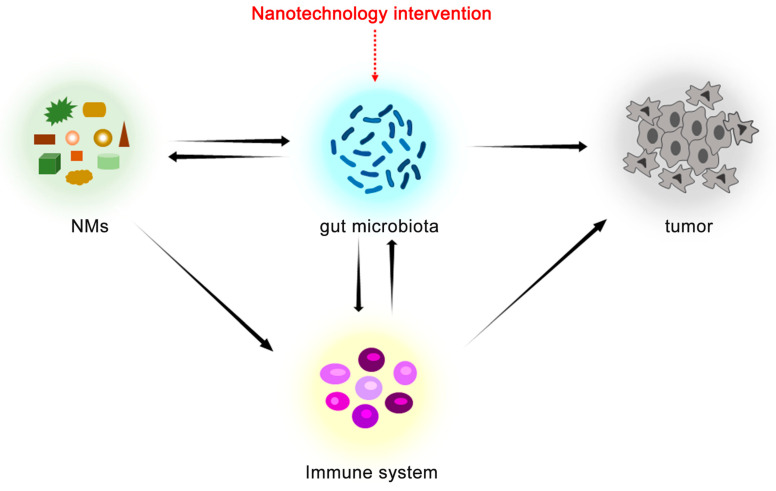
Nanotechnology intervention of gut microbiota for cancer therapy.

A full understanding of nano–bio interactions may lead to safer and more efficacious nano therapeutics. Although NMs demonstrate potent antitumor activity in preclinical models, the behavior of NMs in human cancer remains largely unexplored due to the complexity of the interactions between NMs and the organisms. This complexity is reflected by the fact that NMs also involve the immune system and gut microbiota in the anti-tumor process, and the relationship between them is complex (Figure 4). Nanotechnology can present unique advantages in complex environments by enhancing the selectivity and potency of chemical, physical, and biological approaches through active and passive targeting. Gut microbiota, as a complex ecosystem, can be precisely targeted and regulated by nanotechnology. In addition, due to the mediating role of gut microbiota in NMs and immune system, this strategy can also positively affect the immunity and TME, thereby eliminating tumors. Therefore, targeting gut microbiota is a promising approach in cancer nanomedicine, in which gut microbiota is a key factor affecting its therapeutic effects. However, nanotechnologies for gut microbiota intervention in cancer still face some challenges. Gut microbiota is a delicate and dynamic ecosystem whose homeostasis can be disturbed by external factors, which means that gut microbiota is dynamic and unique to each organism. This requires nanotechnology to be able to target and regulate gut microbiota under its challenging and changing states. At present, however, nanotechnology is still unable to adequately implement these demands, and there are problems to be overcome, such as targeting efficiency and biodistribution, toxicity, and side effects. Overall, nanotechnology is still a nascent field, but that does not prevent it from providing insights into the nano–bio interaction and showing great potential for cancer treatment. In the foreseeable future, nanotechnology that directly targets TME and kills cancer cells, modulates immunity, and regulates gut microbiota may become more effective nanotherapeutic strategies.

In summary, we discussed the interactions of NMs with gut microbiota and their applications in cancer therapy. There is a tight connection between gut microbiota and tumorigenesis, as well as anti-cancer therapy. Nanotechnology-based gut microbiota interventions are being developed and evaluated. NMs engineered by nanotechnology have excellent specificity and local activity, so they are suitable for targeting and regulating gut microbiota. Indeed, nanotechnology for gut microbiota modulation offers promising therapeutic platforms in cancer. We hope that precision medicine for cancer can be achieved through the targeted intervention or precision editing of gut microbiota.

## Data Availability

Not applicable.
